# Clinical Manifestations of Acute COVID-19 in Previously Healthy Pediatric Patients Diagnosed by Rapid Antigen Screening in a Community-Based, Outpatient Primary Care Pediatrics Practice

**DOI:** 10.3390/children11111344

**Published:** 2024-11-01

**Authors:** Stanley Calderwood, Eduardo. L. Montoya, Mandeep Singh Brar

**Affiliations:** 1 PediCenter and Niles Children’s Clinic, Bakersfield, CA 93306, USA; 2 Department of Mathematics, California State University, Bakersfield, CA 93311, USA; emontoya2@csub.edu

**Keywords:** SARS-CoV-2, COVID-19, pandemic, severity of illness, screening, children

## Abstract

Background: The PediCenter and Niles Children’s Clinic provide pediatric primary and urgent care services in central California. We remained open throughout the COVID-19 pandemic, providing scheduled well child-care and sick visits. Methods: Beginning in September 2020, we implemented a COVID-19 screening program. Screening was performed on all patients presenting for care and was made available to patients requiring testing for any purpose. Herein, we provide results from that program, including a description of clinical characteristics of COVID-19 in our patients. Results: Key findings: A total of 11,649 COVID-19 antigen screening tests were performed (age range 0.1 to 17.0, mean 8.7, SD 4.5). In total, 1560 pts. (13.4%) tested positive. Among these, 665 (43%) were asymptomatic, 560 (36%) had mild disease, 318 (20%) had moderate disease, and 17 (1%) had severe disease. No critical cases or transfers to the emergency room were reported. Younger patient age was associated with an increased severity of illness, as was time from the onset of the pandemic. A total of 4446 patients reported no symptoms at the time of screening, 15% of whom tested positive. In total, 7203 patients reported symptoms at the time of testing. Among these, 87.6% tested negative and 12.4% tested positive. Disease severity was similar between these two groups. COVID-19 is generally a mild respiratory tract infection in healthy children. Conclusions: Screening is effective in identifying cases, including asymptomatic cases. Statistical models further revealed associations between patient age, time from the onset of the pandemic, and disease severity.

## 1. Introduction

Beginning in December 2019 and continuing through February 2023, a novel coronavirus, severe acute respiratory syndrome coronavirus 2 (SARS-CoV-2), caused a worldwide pandemic [1]. Infection caused by SARS-CoV-2 is referred to as Coronavirus Infectious Disease 2019 (COVID-19) [2]. A lack of herd immunity and high infectivity rates at the beginning of the pandemic resulted in the rapid spread of the virus, mandating a coordinated Public Health response. In the US, pandemic mitigation efforts including travel restrictions, the quarantining of new arrivals, social distancing, masking, and lockdowns were implemented [3]. These measures were aimed at reducing the opportunity for the virus to circulate in a susceptible population and may have slowed the spread of the virus [4,5], reducing the demand on healthcare systems and allowing time for the development of vaccines and therapeutics [6]; however, they had unanticipated adverse impacts on pediatric patients. These included a decrease in scheduled health maintenance visits [5,7,8], a decrease in vaccination rates [9,10,11], an increase in rates of obesity [12], and reductions in access to emergency [13,14] and subspecialty services [14,15,16,17,18,19,20]. Significant adverse effects on psychosocial health and wellbeing have also been documented [11,21,22]. In New York, for example, pediatric social determinants of health (SDOHs) including access to food, housing, and legal and public health services were all adversely affected by lockdowns, resulting in significantly more emotional and behavioral health concerns [23].

A substantial amount of the literature addresses the clinical manifestations of COVID-19 in adults [24,25] and in pediatric patients with chronic illness [14,20,26,27,28]; however, considerably fewer studies have addressed its clinical manifestations in previously healthy children [29,30]. These studies, along with aggregated data from the CDC, suggest that as many as 40–50% of infections in children may be asymptomatic, while fewer than 1% have severe illness and an even smaller percentage experience long-term sequalae [25,29].

The PediCenter and Niles Children’s Clinic provide pediatric primary and urgent care services to an underserved population in central California. We remained open throughout the COVID-19 pandemic, providing scheduled well child-care and sick visits. Beginning in September 2020, we implemented a COVID-19 screening program to facilitate the safe delivery of patient care. Screening was performed on all patients presenting for care and was also made available upon request for patients requiring testing for any purpose. Herein, we provide results from this screening program and describe the clinical manifestations of acute infection in this patient population.

Although our screening encompassed a large number of pediatric patients, it is important to note that the study participants were specifically those who visited our facilities for care during the study period. Consequently, our findings reflect the characteristics of this group and may not necessarily extend to the general population. This focus allows us to offer detailed insights into the infection dynamics and clinical presentations within a specific, potentially unique community setting.

## 2. Materials and Methods

Following the implementation of the routine screening program in September 2020, all patients presenting for care or requesting testing for any reason were asked to complete a questionnaire identifying symptoms and signs of possible COVID-19 infection. Patients’ vital signs including temperature and oxygen saturation were obtained and a nasal swab for rapid COVID-19 antigen testing was obtained using a commercially available antigen testing kit [Diatrust^TM^, Celltrion, NJ, USA]. Rapid antigen testing has sensitivity of >86% and specificity of >99% (https://ncbi.nim.gov, accessed on 25 October 2024). This is consistent with other reported results in symptomatic adults and children [31,32]. All results were recorded in the patient’s electronic medical record (EMR).

Results of testing were made available to providers prior to patient contact. Patients presenting with symptoms or those who tested positive were placed in isolation. All providers were provided with personal protective equipment (PPE), and universal precautions were employed. Patient rooms were sanitized between patient encounters.

Data presented here were obtained from a retrospective chart review of electronic medical records (EMRs) for all encounters occurring between 1 November 2020 and 31 December 2022. The study protocol was reviewed by an institutional human subjects review committee (HSRC). Since only anonymous “off-the-shelf” data from a retrospective chart review were used and no additional patient contact was planned, the requirement for written informed consent was waived.

Inclusion criteria were a patient age less than or equal to 17 years and those with available COVID-19 antigen test results. To ensure patient anonymity, all encounters were assigned a unique patient encounter number (UPEN) and all other patient identifiers were removed prior to data export. Data exported included patient age (years), sex, date of service, reason for visit, final diagnosis (ICD10 code), temperature (Fahrenheit), pulse oximetry (percent saturation), body mass index (BMI), and COVID antigen test result. 

In our study, patients were classified as having COVID-19 if the antigen test was positive. We employed the severity of illness index recommended by the Centers for Disease Control (CDC) [National Institutes of Health, COVID-19 Treatment Guidelines [https://www.covid19treatmentguidelines.nih.gov/, accessed on 1 January 2023] as follows:

(1) Asymptomatic/presymptomatic: patient reported no symptoms or signs at the time of testing and vital signs were normal.
(2) Mild disease: patient exhibited symptoms or signs of respiratory or gastrointestinal illness with or without fever but with no evidence of lower respiratory tract infection.
(3) Moderate disease: patient had evidence of lower respiratory tract infection.
(4) Severe illness: patient had an oxygen saturation level less than 94%.
(5) Critical illness: patient required transfer to emergency department or hospital admission for a higher level of care.

In addition, we created a seasonal index based on the date of each patient’s visit. This index assigned a value of 1 to any patient who visited during fall 2020, 2 for winter 2021, and so on, through nine seasonal periods ending in fall 2022 (1—fall 2020, 2—winter 2021, 3—spring 2021, 4—summer 2021, 5—fall 2021, 6—winter 2022, 7—spring 2022, 8—summer 2022, 9—fall 2022). The seasonal index was used as a proxy to explain temporal variations in the pandemic’s progression. Although not strain-specific, this approach allowed us to account for general changes over time, reflecting potential shifts in virus strains or public health measures. This method was necessary given that rapid antigen tests, while effective at detecting infection, did not provide data on specific strains.

Subset analysis was performed on the following groups:

(1) All patients who tested positive. (Group 1).
(2) All patients who were symptomatic regardless of test status. (Group 2).

Severity of illness and factors impacting the severity of illness were analyzed using Group 1 patients. A comparison of the severity of symptomatic COVID-19 with other non-COVID-19 infections (defined in our study as symptomatic patients who tested negative for COVID-19) was performed using patients in Group 2. Table 1 provides a description and total numbers of patients in each group.

The primary variables of interest in this study were symptomatic status, test result, age, gender, BMI, seasonal index, and severity level of illness for patients. We considered a *p*-value smaller than 0.05 as statistically significant for all analyses. 

A preliminary univariate analysis was conducted among patients who tested positive to investigate potential associations between severity of illness and patient age, gender, time from the onset of the pandemic, and BMI. A chi-square test found no significant association between gender and severity of illness ($X^{2}=$ 8.68, df = 8, *p*-value = 0.37). For the purpose of a chi-square test, to evaluate the effect of age, we classified patients into three qualitative age groups: infants (birth to 1 year), children (1 year through 12 years), and adolescents (13 years to 17 years). A chi-square test found a significant association between these age groups and severity of illness ($X^{2}=$ 38.602, df = 8, *p*-value < 0.001). To assess the effect of BMI, we categorized patients into three groups: healthy (BMI < 28), mildly obese (BMI from 28 to 34), and obese (BMI greater than 35). We were not able to confirm a statistically significant association between BMI and severity of illness; however, there was a trend towards increasing severity in obese patients. ($X^{2}=$ 18.558, df = 12, *p*-value = 0.10).

A multivariate analysis was conducted using two ordinal logistic regression models to assesses the following issues regarding severity levels:

Model 1: Among patients who tested positive, are levels of severity associated with a patient’s age, gender, and seasonal index?

Model 2: Among all symptomatic patients, do levels of severity differ among the positive and negative patients while accounting for variables that may significantly affect the level of severity (age, gender, seasonal index)? 

Note that we evaluated the impact of gender, age, BMI, and seasonal index on the severity of illness in patients who tested positive (Model 1). In addition, we compared the severity of illness in symptomatic patients who tested positive against those who tested negative (Model 2). Furthermore, note that the dependent variable, level of severity, followed an ordinal distribution with distinct levels representing four different levels of severity (asymptomatic, mild, moderate, and severe). Ordinal logistic regression allowed one to investigate the likelihood of each severity level occurring based on a set of independent variables. In this analysis, we modeled the cumulative odds of each severity level using the logit transformation. Specifically, we examined how the log-odds of the cumulative probability of a specific severity level were linearly related to the independent variables. This approach enabled us to explore the factors associated with different severity levels [33].

To determine which variables significantly affected the likelihood of each severity level occurring, forward model selection was used [34]. This method was used to build each model, using a likelihood ratio test as the selection criterion [35]. The estimated model coefficients, standard errors, two-sided *p*-values, exponentiated model coefficients (OR), and 95% confidence intervals (CIs) for the exponentiated estimated regression coefficients are given in tables for Model 1 and 2, respectively.

## 3. Results

A total of 11,649 COVID-19 antigen screening tests were performed. Patients ranged in age from 0.1 to 17.0 years, (mean 8.7 y, SD 4.5 y). A total of 1560 pts. (13.4%) tested positive (Group 1). Among these, 665 (43%) were asymptomatic, 560 (36%) had mild disease, 318 (20%) had moderate disease, and 17 (1%) had severe disease. 

There were 4446 patients who reported no symptoms and presented for screening, only 15% of whom tested positive. These patients were included in descriptive statistics but were not included in any other statistical analysis. 

There were 7203 patients who reported symptoms consistent with COVID-19 at the time of testing (Group 3). Among these, 87.6% tested negative and 12.4% tested positive. 

Brief descriptions of all variables used in this study are provided in Table 2.

The first quartile, median, mean, and third quartile of the quantitative data, as well as the frequency counts and percentage for levels of the observed qualitative data, for the primary variables of interest are presented in Table 3.

The reasons for visits (chief complaints) of study participants are presented in Table 4. Reason for visit was recorded verbatim in a text field in the EMR; therefore, for ease and convenience of the presentation of data, chief complaints were categorized as predominantly respiratory symptoms (nasopharyngitis, cough, wheezing, chest pain, or shortness of breath), predominantly gastrointestinal symptoms (nausea, vomiting, diarrhea, abdominal pain, or loss of appetite), constitutional symptoms (fever, headache, body aches, or fatigue without respiratory or gastrointestinal symptoms), skin rash with no other symptoms, and screening only (patients who presented without symptoms, requesting testing for any purpose).

Final diagnoses for patients with severe disease (those with oxygen saturation <94% on presentation) are presented in Table 5. The final diagnosis was based on the ICD-10 code assigned by the healthcare provider at the time of the visit.

For a given model result, any estimated regression coefficient is interpreted while keeping the other variables in the model constant. To identify the most significant predictors for our models, we employed an AIC forward stepwise regression approach, optimizing for the best balance between model complexity and predictive accuracy. Following the initial selection via the AIC, we further refined our model using Maximum Likelihood Estimation tests to ensure that only statistically significant variables were retained.

Table 6 displays the results from Model 1 analysis, which evaluated the impact of age and seasonal index on the severity of illness among patients who tested positive for COVID-19. We found that gender and BMI were not significant predictors in this model. The effect of the seasonal index conveys that the odds of being in a higher severity category increase by 35% (95% CI: 25.9% to 44.6%) for each progression from one season to the next. The effect of the patient’s age suggests that for every additional year of age, the odds of being in a higher severity category decrease by 5% (95% CI: 3.5% to 7.0%). 

Results for model 2 are displayed in Table 7. Model 2 considered all symptomatic patients to assess whether levels of severity differ among those who tested positive and negative for COVID-19, while accounting for variables that may significantly affect the level of severity of a patient. The results showed that patients who tested positive for COVID-19 have 33.20% higher odds (95% CI: 19.00% to 48.96%) of being in a higher severity level compared to those who tested negative. Additionally, for each additional year of a patient’s age, the odds of being in a higher severity level decrease by about 7.22% (95% CI: 6.46% to 7.98%). As we progress from one season to the next, the odds of being in a higher severity level increase by 41.42% (95% CI: 37.86% to 45.06%). These findings provide evidence at the significance level of 0.05 that the severity differs among those who tested positive and negative for COVID-19, even after accounting for factors that significantly affect the level of severity. 

## 4. Discussion

SARS-CoV-2 infects cells by adhering to cell membrane angiotensin-converting enzyme 2 (ACE-2) receptors [24]. The ACE-2 receptor is widely distributed on endothelial cells, the renal epithelium, and respiratory epithelial cells, particularly in the lower respiratory tract [36]. Children are known to have a significantly reduced concentration of ACE-2 receptors on their lower respiratory tract epithelial cells and would therefore be predicted to have a lower severity of illness than older adults [37]. This may, in part, explain the mild symptom severity in the majority of our patients. Our findings are consistent with reports from the CDC and other investigators [11,30] which suggest that for most previously healthy pediatric patients, COVID-19 is a very mild infection, with a significant percentage having no symptoms at the time of diagnosis. 

We note that our findings from Model 1 show an unexpected association between age and severity of illness in that for every additional year of age, the odds of a patient being in a higher severity category decrease by 5%. Although the odds ratio is close to 1, research has demonstrated that even small effect sizes can have substantial public health implications when applied to large populations (Carey et al., 2023). This unique observation suggests that the relationship between age and severity in pediatric patients may differ from that in older populations, and this warrants further investigation. Recent studies have suggested that SARS-CoV-2 can cause bronchiolitis in infants [38,39]. A final diagnosis of bronchiolitis was recorded in 320 (2.7%) of our total study population and in 11.8% of our patients with severe disease. This may, in part, explain our finding of the increased risk of severe disease in younger patients. Additional studies are planned to further investigate this association.

It should be noted that 52.9% of our patients with severe infection had a final diagnosis of nasopharyngitis (common cold). Obtaining accurate oxygen saturation by pulse oximetry can be difficult in children, especially younger children who are often crying and non-compliant. Our statistical analysis plan did not allow for the re-assignment of severity of illness after data extraction; therefore, our finding of 1% of patients with severe infection is likely a significant overestimation. 

While we observed a trend towards an increasing severity of illness in obese patients (*p* = 0.1), the association did not achieve statistical significance. Note that our analysis did not find evidence of a significant relationship at our chosen significance level between BMI and the severity of illness in Model 1; however, our analysis was constrained by the limited availability of BMI data, which were lacking for approximately 86% of patients, perhaps impacting the ability of our study to confirm an association as reported by other authors [40,41,42,43].

Our testing consisted solely of rapid antigen detection by immunofluorescent assay, which does not identify viral strain. For that reason, we included an analysis of the time from the onset of the pandemic to the time of testing, which was intended as a surrogate marker for the impact of the dominant circulating strain on the severity of illness. Our data show a statistically significant association between the time from the onset of the pandemic and severity of illness (seasonal index), with patients experiencing more severe symptoms as more time from the beginning of the pandemic passed. This is most likely explained by the fact that the original SARS-CoV-2 variant, the alpha variant, was displaced as the predominant strain beginning in December of 2020 by the Delta variant, and Delta was displaced as the predominant strain in about December of 2021 by the Omicron variant, both of which were documented to be more contagious and to cause more severe illness than the original Alpha variant [44,45]. Our data show a statistically significant association between the time from the onset of the pandemic and severity of illness (seasonal index), supporting the notion of these strains being more contagious and severe. Screening proved effective in identifying patients with asymptomatic/presymptomatic infection, facilitating the safe continuation of outpatient services, when combined with appropriate isolation and universal precautions. The deleterious effects of restricted access to healthcare are now well documented, and we hope our experience may help guide policy decisions during future pandemics. Screening, universal precautions, and PPE can be safely and effectively utilized in a primary care setting to facilitate the continued provision of care. 

Since SARS-CoV-2 is transmitted by droplets, it is unclear whether asymptomatic patients can transmit the infection [46,47]; notwithstanding, asymptomatic patients may be a vector for infection [46]. For this reason, all patients who tested positive, regardless of symptoms, were placed in isolation rooms in clinic and were instructed to isolate at home, and family contacts were instructed to quarantine as per CDC guidelines.

Amongst patients who reported symptoms at the time of diagnosis, those who tested negative and were assumed to have an infection other than COVID-19 did not differ significantly demographically from patients who tested positive for COVID-19. Although an infection was not documented or confirmed in all patients who tested negative, reasons for visit and final diagnoses were similar in the two groups. Results from Model 2 suggest that the severity of illness score for symptomatic patients who tested positive for COVID-19 was higher than for symptomatic patients who tested negative. An obvious criticism of this analysis is that patients who were asymptomatic/presymptomatic and who tested positive were excluded from this subset analysis, skewing the severity of illness towards a higher level. Notwithstanding, symptomatic patients with COVID-19 need to be evaluated and monitored carefully for severe disease. 

A limitation of our data is that the data relied on the self-reporting of symptoms; therefore, it is possible that patients presenting for screening who required clearance to participate in school or other activities may have under-represented their symptoms, skewing the severity of illness index towards milder infections. This concern is partially offset by the fact that to be designated asymptomatic, patients also needed to have normal vital signs and normal oxygen saturation. 

Our study only evaluated patients at the time of encounter for screening, and we do not have follow-up data to address what percentage of patients went on to develop symptoms. Likewise, we do not have long-term follow-up data to evaluate risk of long COVID [48] or complications including multisystem inflammatory syndrome associated with COVID (MIS-C) [49]. Additional studies are currently planned to address these issues.

## 5. Conclusions

Our purpose in conducting this study and presenting our findings is to demonstrate that adherence to well-established universal precautions, along with use of PPE and routine screening, as implemented in our outpatient practice, can be easily accomplished and can be effective in facilitating the safe continuation of office practice during future pandemics, a desirable outcome if the adverse impacts of lockdowns, as previously enumerated, are to be avoided or mitigated. Further, our study indicates that clinical research can be conducted in a non-academic, outpatient setting and that such research can contribute to the state of knowledge regarding illnesses that impact the general pediatric population, the majority of which is cared for in the community, outside of university-affiliated hospitals and clinics. 

Overall, our analysis provides valuable insights into the association of variables such as age, gender, seasonal index, and BMI with the severity of illness. These data suggest that COVID-19 in children is generally a mild infection, with a substantial proportion reporting no symptoms at the time of diagnosis, a fact that was not well known or anticipated at the onset of the pandemic. Only 1% had severe illness as per the CDC severity rating, and no patient required transfer to ER or hospital admission. Younger patient age was a statistically significant risk factor for increasing severity of illness, as was time from the onset of the pandemic. While there was no significant association found between BMI and severity in our dataset, this may warrant further investigation given the limited availability of BMI data in our study.

Despite the generally mild severity of COVID-19 in children, the disease burden, including school and work absenteeism, hospital admission, long-term sequelae, and even death, are still substantial, so in our practice, we continue to advocate for COVID-19 vaccination in all children older than 6 months of age [50].

## Figures and Tables

**Table 1:** Categorization of the study population into symptomatic and asymptomatic groups, detailing those who tested positive and negative for COVID-19. Group 1 includes all patients who tested positive (N_1_ + N_3_ = 1560), covering both symptomatic (N_1_ = 895) and asymptomatic (N_3_ = 665) cases. Group 2 consists of all symptomatic patients (N_1_ + N_2_ = 7203), irrespective of their test results.

	COVID-19 Positive	COVID-19 Negative	Total
Symptomatic	N_1_ = 895	N_2_ = 6308	Group 2: 7203
Asymptomatic	N_3_ = 665	N_4_ = 3781	
Total	Group 1: 1560		

**Table 2:** Description of variables.

Gender	The gender identity of the patient, as self-reported during their visit.
Date of Service	The specific date when the patient received medical services.
Given Reason for Visit	The primary health concern or issue that prompted the patient’s visit, as described by the patient.
International Classification of Diseases (ICDs)	Codes that represent the patient’s diagnosis. These codes are standardized by the World Health Organization (WHO) and used globally to classify diseases and health conditions.
COVID-19 Testing Result	The outcome of the patient’s test for the COVID-19 virus is typically recorded as positive, negative, or pending.
Age	The patient’s age in years at the time of service.
Temperature	The patient’s body temperature at the time of the visit, measured in degrees Fahrenheit.
Pulse Oximetry	A measure of the patient’s oxygen saturation level indicates how much oxygen is in the blood as a percentage.
BMI and Standardized BMI	BMI is computed as weight in kilograms divided by the square of height in meters. Standardized BMI represents the number of standard deviations a patient’s BMI is from the mean BMI value of a reference population, based on standardized growth tables.
Symptom Status	Classifies each patient based on the presence or absence of illness symptoms, denoted as asymptomatic or symptomatic.
Severity of Illness	Defines the level of illness severity in patients (1—asymptomatic, 2—mild, 3—moderate, and 4—severe) according to the Clinical Spectrum of SARS-CoV-2 Infection criteria.
Severity Category	Further categorizes the illness severity of symptomatic patients into two groups: non-severe (for mild and moderate illness) and severe.
Seasonal Index	Represents the sequence of seasons by year covering the study period. Patients who visited during the first semester of the data collection are assigned a ‘1’, those in the second semester are assigned a ‘2’, and so forth, marking the seasonal chronological progression of visits.

**Table 3:** Numerical summaries for the data. Severity of illness is an ordinal qualitative variable but a numerical summary of the numerical representation is provided solely for the purpose of providing a numerical summary.

	1st Quartile	Median	Mean (SD)	3rd Quartile
Age	5	8	8.70 (4.54)	12
BMI	16	20	21.90 (7.80)	26
Severity of Illness	1	2	1.72 (0.65)	2
Standardized BMI	−0.12	1.06	0.74	1.82
Seasonal period	6	6	6.61 (1.52)	8
	Frequency count			
Gender	Female: 5559 Male: 5885 Other: 205			
COVID-19 Testing result	Negative: 10,089 Positive: 1560			

**Table 4:** Reasons for visits for study subjects, with percentage of patients experiencing each as predominant symptom.

Reason for Visit	Percentage
Respiratory Symptoms	41%
Gastrointestinal Symptoms	7%
Constitutional Symptoms	23%
Skin Rash	1%
Screening	28%

**Table 5:** Final diagnosis (ICD-10 code) and percentage of patients with that diagnosis for patients with severe disease by CDC criteria (oxygen saturation of <94% by pulse oximetry at presentation).

Broncholitis	17.65%
Bronchitis	11.77%
Croup	5.88%
Nasopharyngitis	52.94%
Viral Pneumonia	11.77%

**Table 6:** Model 1 summary.

Independent Variables	Estimated Regression Coefficients	Standard Errors	*p*-Value	OR	95% Confidence Intervals for OR
Seasonal index	0.298	0.035	<0.001	1.348	(1.259, 1.446)
Age	−0.054	0.010	<0.001	0.947	(0.930, 0.965)

**Table 7:** Model 2 summary. Note: “Gender (Other)” is used as the reference category for gender comparisons.

Independent Variables	Estimated Regression Coefficients	Standard Errors	*p*-Value	OR	95% Confidence Intervals for OR
Testing result (+)	0.292	0.057	<0.001	1.339	(1.196, 1.498)
Seasonal index	0.345	0.013	<0.001	1.412	(1.376, 1.448)
Age	−0.075	0.004	<0.001	0.928	(0.920, 0.936)
Gender (male)	0.031	0.037	0.412	1.031	(0.958, 1.109)
Gender (female)	−0.488	0.145	<0.001	0.614	(0.461, 0.814)

## Data Availability

Original data tables can be made available upon request to the corresponding author.
